# Characterisation of a Tip60 Specific Inhibitor, NU9056, in Prostate Cancer

**DOI:** 10.1371/journal.pone.0045539

**Published:** 2012-10-08

**Authors:** Kelly Coffey, Timothy J. Blackburn, Susan Cook, Bernard T. Golding, Roger J. Griffin, Ian R. Hardcastle, Lorraine Hewitt, Kety Huberman, Hesta V. McNeill, David R. Newell, Celine Roche, Claudia A. Ryan-Munden, Anna Watson, Craig N. Robson

**Affiliations:** 1 Solid Tumour Target Discovery Laboratory, Newcastle Cancer Centre, Northern Institute for Cancer Research, Newcastle University, Newcastle upon Tyne, Tyne and Wear, United Kingdom; 2 Drug Discovery and Imaging Group, Newcastle Cancer Centre, Northern Institute for Cancer Research, Newcastle University, Newcastle upon Tyne, Tyne and Wear, United Kingdom; 3 OSI Pharmaceuticals, Inc, Farmingdale, New York, United States of America; 4 Research and School of Chemistry, Newcastle University, Newcastle upon Tyne, Tyne and Wear, United Kingdom; Innsbruck Medical University, Austria

## Abstract

Tip60 (KAT5) is a histone acetyltransferase (HAT enzyme) involved in multiple cellular processes including transcriptional regulation, DNA damage repair and cell signalling. In prostate cancer, aggressive cases over-express Tip60 which functions as an androgen receptor co-activator via direct acetylation of lysine residues within the KLKK motif of the receptor hinge region. The purpose of this study was to identify and characterise a Tip60 acetylase inhibitor. High-throughput screening revealed an isothiazole that inhibited both Tip60 and p300 HAT activity. This substance (initially identified as 4-methyl-5-bromoisothiazole) and other isothiazoles were synthesised and assayed against Tip60. Although an authentic sample of 4-methyl-5-bromoisothiazole was inactive against Tip60, in an *in vitro* HAT assay, 1,2-bis(isothiazol-5-yl)disulfane (NU9056) was identified as a relatively potent inhibitor (IC_50_ 2 µM). Cellular activity was confirmed by analysis of acetylation of histone and non-histone proteins in a prostate cancer cell line model. NU9056 treatment inhibited cellular proliferation in a panel of prostate cancer cell lines (50% growth inhibition, 8–27 µM) and induced apoptosis via activation of caspase 3 and caspase 9 in a concentration- and time-dependent manner. Also, decreased androgen receptor, prostate specific antigen, p53 and p21 protein levels were demonstrated in response to treatment with NU9056. Furthermore, pre-treatment with NU9056 inhibited both ATM phosphorylation and Tip60 stabilization in response to ionising radiation. Based on the activity of NU9056 and the specificity of the compound towards Tip60 relative to other HAT enzymes, these chemical biology studies have identified Tip60 as a potential therapeutic target for the treatment of prostate cancer.

## Introduction

Histone acetylation and deacetylation are key events in the regulation of chromatin structure. Histone acetyltransferases (HATs) catalyze the addition of acetyl groups to the ε-amino terminus of lysine residues within histones. Acetylation results in an open chromatin structure by removing positive charges from histones, thus inducing protein conformational changes, which allows transcriptional machinery to access the DNA and promote transcriptional activity. Histone deacetylases (HDAC) oppose this process by promoting a closed chromatin structure, which is transcriptionally repressed. Furthermore, histone acetylation marks can function as docking sites for other proteins to interpret the ‘histone code’; for example, the tripartite motif containing 24 (TRIM24) was recently described as a ‘reader’ protein, which recognises both unmodified histone H3 at lysine 4 and histone H3 acetylated at lysine 23 on the same histone tail resulting in increased gene expression [1]. In addition, non-histone proteins such as p53 [2], [3], ataxia telangiectasia mutated (ATM) [4] and androgen receptor (AR) [5], [6] can also be acetylated resulting in altered protein activity. Hence, protein acetylation and deacetylation can have significant effects on cell function, and for cells to maintain normal growth and differentiation it is important that these two functions maintain equilibrium. In support of this concept, HDAC inhibitors have been found to have wide ranging cellular effects and clinical activity in leukaemia [7], [8], with Vorinostat (SAHA) being approved for clinical use in this disease. Modulation of histone acetylation clearly has therapeutic potential.

Tip60, recently renamed KAT5, is a member of the MYST family of HAT enzymes first identified in 1996 [9]. Since then many cellular functions have been found to use this protein. Loss of Tip60 results in impaired DNA repair, as this HAT is activated in response to ionising radiation (IR), causing acetylation of histones and activation of p53 and ATM [4]. Inhibition of Tip60 should therefore sensitise cells to DNA damaging agents used as cancer therapeutics. Tip60 also functions in the NF-κB pathway, via interactions with B-cell CLL/lymphoma 3 (BCL-3) [10] and cAMP-dependent signalling [11]. Furthermore, Tip60 can function as a co-activator for a number of steroid hormone receptors including the AR, which is involved in the development and progression of prostate cancer (CaP). Studies have shown that AR can be acetylated by a number of HAT enzymes, including p300, p300/CBP-associated factor (PCAF) and Tip60, to increase its transcriptional activity [6], [12]. AR acetylation is thought to regulate the recruitment of co-activators to the transcriptional machinery of androgen responsive genes [13]. Additionally, Tip60 is functionally up-regulated in clinical CaP specimens and expression correlates with disease progression [14]. In contrast, one report suggested that Tip60 is required to express the tumour metastasis suppressor KAI1 in CaP cell lines, suggesting that Tip60 is a tumour suppressor [15]. Similarly, a Tip60 gene knockout study proposed Tip60 as a haplo-insufficient tumour suppressor at pre and early-tumoral stages of lymphoma, breast and head and neck cancers [16]. However, studies on clinical prostate specimens contradict this suggestion and support Tip60 as an oncogene in CaP [13], [17]. Thus, targeting the acetylase activity of Tip60 could be a useful therapeutic strategy in CaP.

A small number of HAT inhibitors have been reported. Coupling a histone H3 peptide to CoA to form a bisubstrate inhibitor of HAT activity has been described; however, the compound has poor cell membrane permeability [18]. The natural products anacardic acid and garcinol are HAT inhibitors that are cell permeable; they sensitise cells to IR, which could be useful as a combination therapy for cancer treatment. Other inhibitors of HAT function include α-methylene butyrolactones [19], benzylidene acetones [20] and alkylidene malonates [21]. More recently, isothiazolones, which covalently bind to the HAT active site thiol, have been described as an effective starting point for molecular modelling-based approaches for generating more potent and specific inhibitors [22]–[24]. In the current study we employed a high throughput screening approach to identify selective inhibitors of Tip60. Based on the lead molecule, structurally related compounds were generated and tested for HAT inhibition and Tip60 specificity in order to identify a molecular tool for studies in cell line models of CaP.

## Results

### High Throughput Screen (HTS) and Hit Validation

**Figure 1:**
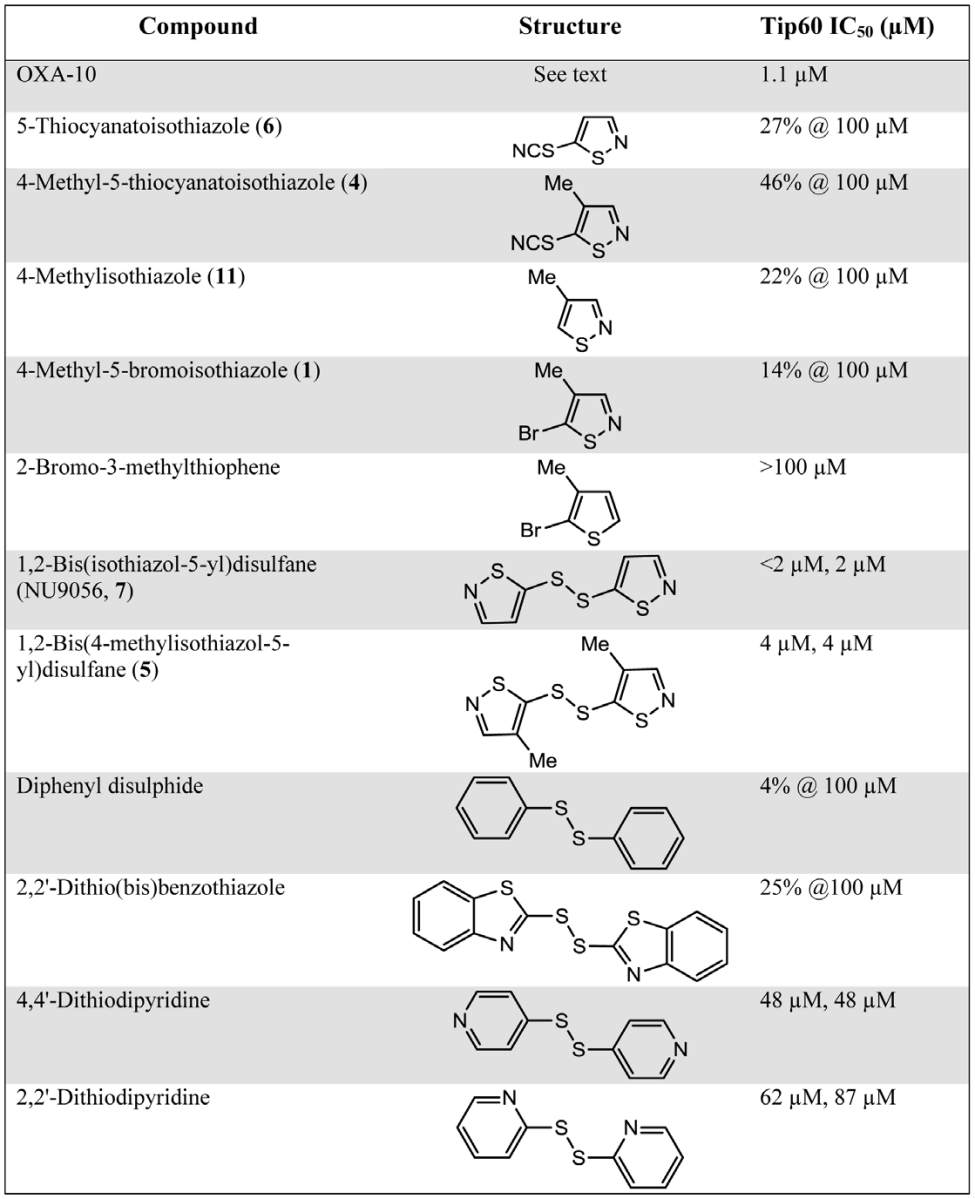
Table of Tip60 IC_50_ values of isothiazoles and related compounds. To assess the activity against Tip60 HAT, *in vitro* HAT assays using ^3^H acetyl-CoA were carried out using histones as substrates. Assays were performed in quadruplicate and repeated twice. For compounds producing >50% inhibition at 100 μM, IC_50_ values were calculated. Individual IC_50_ values are presented. For other compounds the % inhibition at 100 μM is presented.

**Figure 2:**
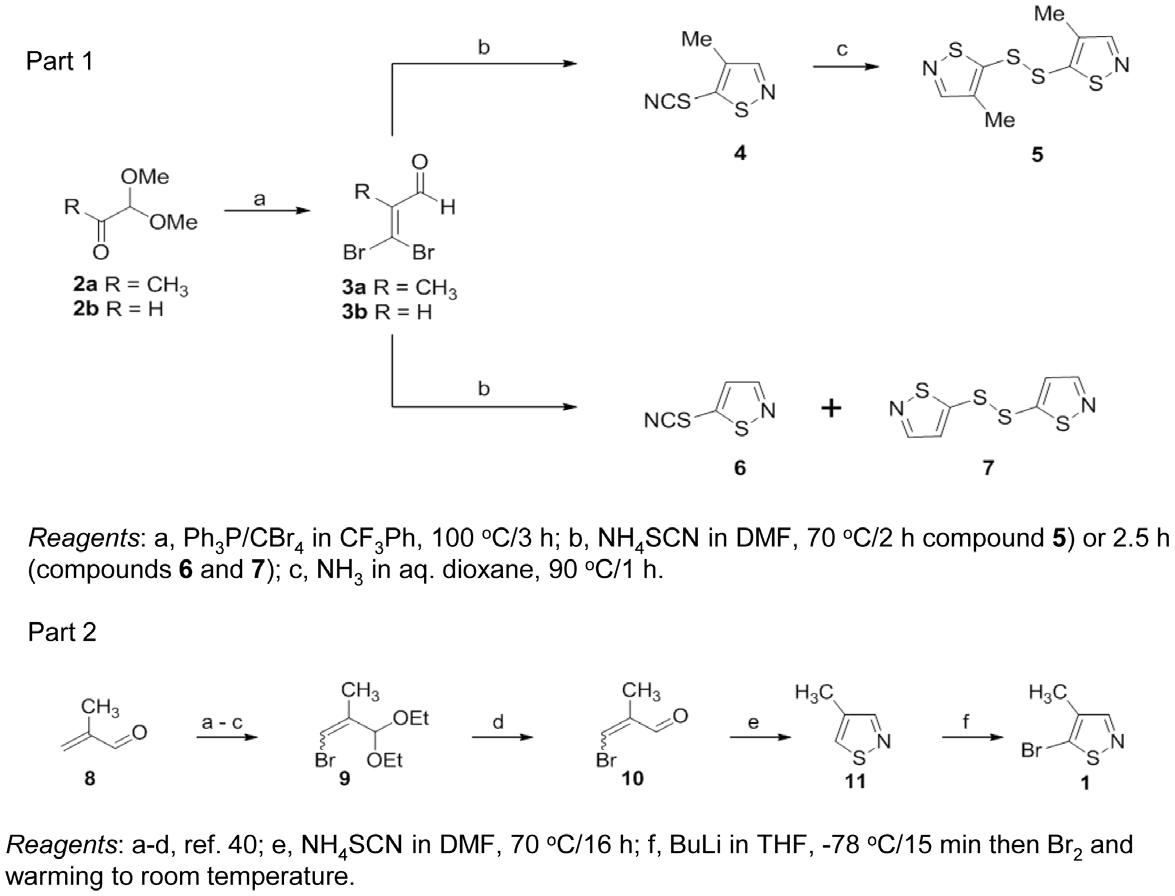
Chemical synthesis of Tip60 inhibitors. Part 1 - Synthesis of compounds **4–7**. Part 2 - Synthesis of Compounds **1** and **11**.

A high throughput screening campaign for Tip60 inhibitors was conducted at OSI Pharmaceuticals Ltd. Assays based on the ALPHA™ screen and DELFIA™ formats were developed and used to screen a structurally diverse compound collection (∼80,000 members). A number of hits were identified from the primary ALPHA™ screen. However, most of these did not show significant activity in the secondary screen. A single compound OXA-10 (initially identified as 4-methyl-5-bromoisothiazole, **1**), was identified as a hit in both screens and the activity was replicated with repurchased material. Repurchased OXA-10 showed activity against Tip60 (IC_50_ 1.1 µM - Figure 1) and p300 (IC_50_ 2.7 µM) (Table 1), but not other histone acetyltransferases, e.g. PCAF and GCN5 (IC_50_>100 µM). LC-MS analysis of the repurchased sample of OXA-10 indicated the presence of ∼80% 4-methyl-5-bromoisothiazole **1**, but showed that it contained ∼20% of an unknown impurity. In order to validate **1** as the active inhibitor of Tip60 in OXA-10, the synthesis of **1** was undertaken, along with analogues, in order to develop structure activity relationships. Details of the chemical synthesis (Figure 2) can be found in Supplementary Information.

**Table 1:** Tip60 IC_50_ values of isothiazoles and related compounds.

Compound	Structure	Tip60 IC_50_ (µM)
OXA-10	See text	1.1 µM
5-Thiocyanatoisothiazole (**6**)	Figure S8	27% @ 100 µM
4-Methyl-5-thiocyanatoisothiazole (**4**)	Figure S9	46% @ 100 µM
4-Methylisothiazole (**11**)	Figure S10	22% @ 100 µM
4-Methyl-5-bromoisothiazole (**1**)	Figure S11	14% @ 100 µM
2-Bromo-3-methylthiophene	Figure S12	>100 µM
1,2-Bis(isothiazol-5-yl)disulfane (NU9056, **7**)	Figure S13	<2 µM, 2 µM
1,2-Bis(4-methylisothiazol-5-yl)disulfane (**5**)	Figure S14	4 µM, 4 µM
Diphenyl disulphide	Figure S15	4% @ 100 µM
2,2′-Dithio(bis)benzothiazole	Figure S16	25% @100 µM
4,4′-Dithiodipyridine	Figure S17	48 µM, 48 µM
2,2′-Dithiodipyridine	Figure S18	62 µM, 87 µM

To assess the activity against Tip60 HAT, *in vitro* HAT assays using ^3^H acetyl-CoA were carried out using histones as substrates. Assays were performed in quadruplicate and repeated twice. For compounds producing >50% inhibition at 100 µM, IC_50_ values were calculated. Individual IC_50_ values are presented. For other compounds the % inhibition at 100 µM is presented.

**Table 2:** IC_50_ values of 1,2-bis(isothiazol-5-yl)disulfane (7) and related compounds towards HATs.

Compound	HAT IC_50_ (µM)			
	Tip60	p300	PCAF	GCN5
1,2-Bis(isothiazol-5-yl)disulfane(NU9056, **7**)	<2, 2	60, 58	30, 36	>100
5-Thiocyanato-isothiazole (**6**)	>100	–	–	–
1,2-Bis(4-methylisothiazol-5-yl)disulfane (**5**)	4, 4	58, 43	33, 25	>100
OXA-10	1.1	2.7	>100	>100
ICR CCT129182	1, 0.9	31, 28	23, 30	>100
AE562	0.8, 0.8	1.8, 2	>100	>100

To assess the activity against Tip60 HAT, *in vitro* HAT assays using ^3^H acetyl-CoA were carried out using histones as substrates. Assays were performed in quadruplicate and repeated twice. Individual IC_50_ values are presented.

### Specificity of NU9056 for Tip60 Acetyltransferase

NU9056 (**7**), as well as compounds **5** and **6,** were tested for *in vitro* activity against a panel of recombinant HAT enzymes, including p300, PCAF and GCN5, to determine whether they show greater specificity towards Tip60 than compound **1**. ICR CCT129182, which has been shown to have inhibitory activity against p300 and PCAF, was also tested [24]. A number of compounds were found to inhibit the activity of Tip60 at low micromolar concentrations (Figure 1; Supplemental Figure S1). However, specificity towards Tip60 over other HAT enzymes tested was found to be greatest with compound **7** (NU9056) as shown in Table 1 (16.5-, 29- and >50-fold for selectivity for Tip60 over PCAF, p300 and GCN5, respectively).

### NU9056 Inhibits Protein Acetylation in Prostate Cancer Cell Lines

**Figure 3:**
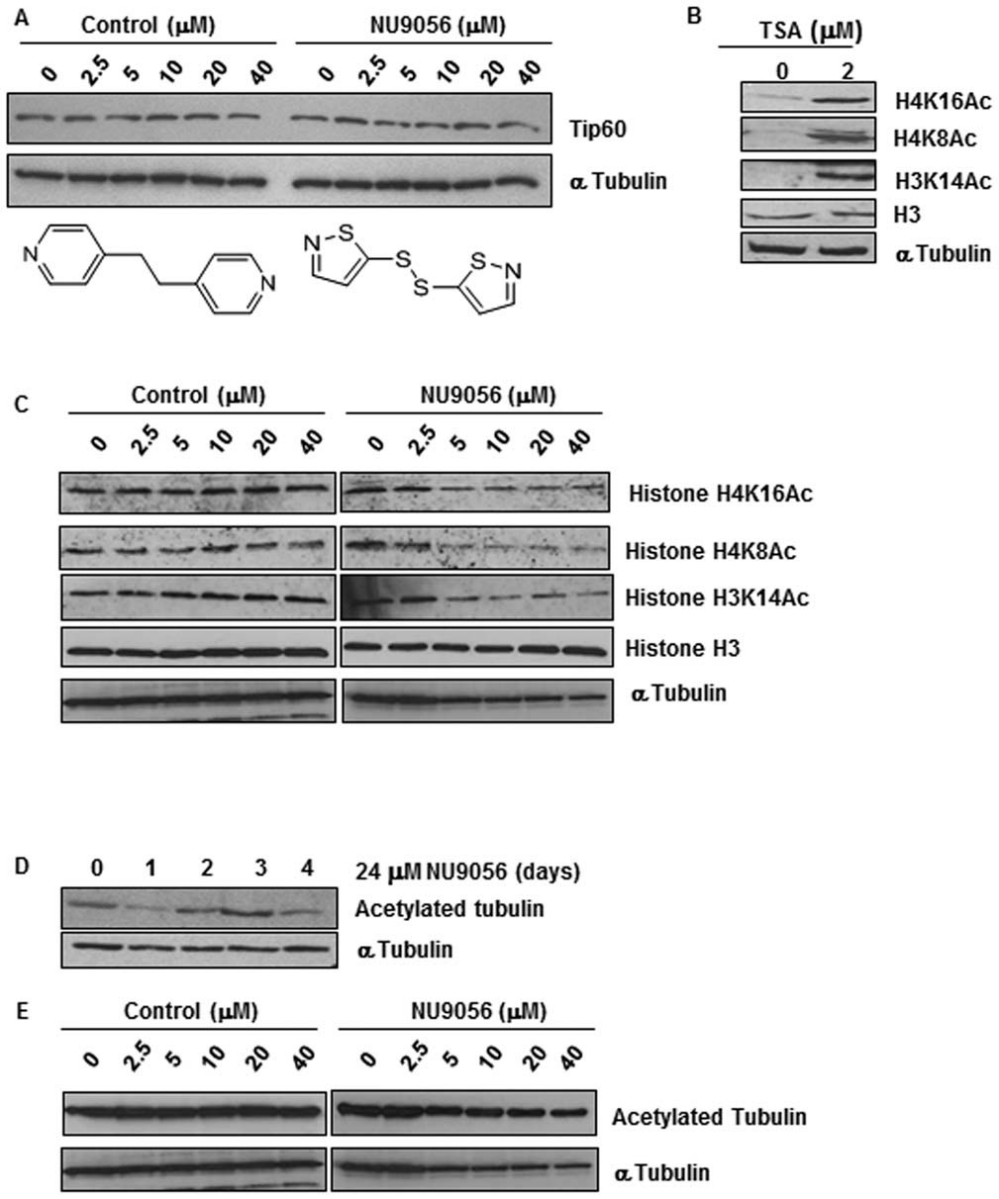
NU9056 inhibits protein acetylation in prostate cancer cell lines. LNCaP cells were treated with increasing concentrations of NU9056 or the control compound, 1,2 bis(4-pyridyl)-ethane for 24 hours. (A) Levels of Tip60 were assessed by Western blotting. (B) LNCaP cells were treated with 2 µM TSA for 6 hours and levels of histone H4 acetylated-lysine 16, histone H4 acetylated-lysine 8 and histone H3 acetylated lysine 14 were assessed by Western blotting. LNCaP cells were treated with increasing concentrations of NU9056 or the control compound for 2 hours, then treated with the HDAC inhibitor TSA (2 µM) for a further 4 hours. (C) Levels of histone H4 acetylated-lysine 16, histone H4 acetylated-lysine 8 and histone H3 acetylated lysine 14 were assessed by Western blotting. (D) LNCaP cells were treated with 24 µM NU9056 over 4 days and the levels of acetylated tubulin were assessed by Western blotting. (E) LNCaP cells were treated with increasing concentrations of NU9056 or the control compound for 2 hours, then treated with the HDAC inhibitor TSA (2 µM) for a further 4 hours. Levels of acetylated tubulin were assessed by Western blotting. Alpha-tubulin was used as a loading control. Representative blots are shown for duplicate experiments.

Tip60 acetylates histone proteins, specifically histones H4 and H2A, in a nucleosomal context [25]. Furthermore, *in vitro* studies have demonstrated that Tip60 can also acetylate core histones H2A (Lys 5), H3 (Lys 14) and H4 (Lys 5, Lys 8, Lys 12 and Lys 16) [26], [27]. To test whether NU9056 could inhibit acetylation of endogenous proteins targeted by Tip60, LNCaP cells were used as these are a representative model of androgen dependent CaP. The acetylation status of histone H4 at lysine 8 (H4K8) and 16 (H4K16) and histone H3 at lysine 14 (H3K14) was investigated in these cells by Western blotting. In addition, the level of Tip60 was assessed following treatment with NU9056 and the control compound, 1,2-bis(4-pyridyl)ethane (Figure 3A) demonstrating that Tip60 levels themselves are unaffected. As the basal levels of these acetylated histone marks are quite low, the HDAC inhibitor trichostatin A (TSA) was introduced to remove the influence of HDAC activity on acetylation in the cellular assay. In the presence of TSA alone, acetylation was increased at H4K8, H4K16 and H3K14 (Figure 3B), consistent with previous reports [28]–[30]. Increasing concentrations of NU9056 resulted in decreased levels of acetylated histone H4K16, H3K14 and H4K8, targets for Tip60-mediated acetylation (Figure 3C). Furthermore, the control compound, 1,2-bis(4-pyridyl)ethane, did not affect any of the histone modifications studied.

To define further the HAT inhibitory activity of NU9056, acetylation of the non-histone protein α-tubulin was also investigated. When LNCaP cells were incubated with NU9056 (24 µM) for 24 hours a decrease in acetylated tubulin was observed. However, by 72 hours acetylation had returned to basal levels (Figure 3D). To confirm that this effect was due to NU9056, acetylated tubulin was monitored but no change in levels was observed within 6 hours unlike alterations to histone modifications (Figure 3E). Densitometry for all Western blots is shown in Supplemental Figure S2.

### NU9056 Inhibits Cell Growth

**Figure 4:**
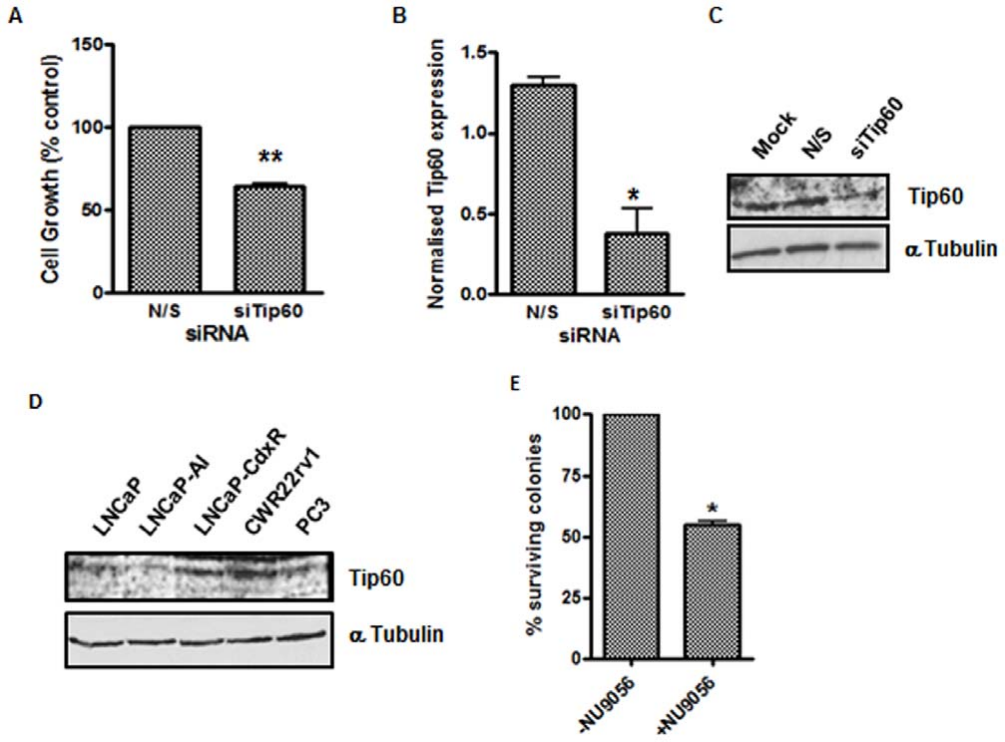
Knockdown of Tip60 reduces proliferation in LNCaP cells. (A) To confirm that inhibition of Tip60 can reduce cellular proliferation 2.5 nM siRNA specifically targeted against Tip60 in LNCaP cells or a non-silencing control was used. Proliferation was determined by sulforhodamine B (SRB) assays at 3 control proliferation doubling times after siRNA transfection in normal growth media. To confirm Tip60 knockdown, RNA was collected at 96 hours from a parallel experiment and assessed for Tip60 expression using (B) real-time PCR and (C) Western blotting. (D) Tip60 levels in prostate cancer cell lines were assessed by Western blotting. (E) Prostate cancer cell survival was assessed by treating LNCaP cells with 24 µM NU9056 for 24 hours then plating at varying cell densities (3×10^3^, 1.6×10^4^ and 3×10^4^) and allowing colonies to form over 2 weeks. Colonies were then fixed with Carnoy’s fixative and stained with crystal violet. Colonies were then counted and colony forming efficiency calculated. The mean of 3 experiments ± standard deviation is shown on bar charts. *p-value <0.05.

We observed that knockdown of Tip60 in LNCaP cells resulted in an inhibition of cell proliferation by approximately 33% (p-value = 0.0019) (Figure 4A). Efficient knockdown of Tip60 mRNA in these cells was confirmed by real-time PCR (Figure 4B, 70% knockdown; p-value = 0.0313) and Western blotting (Figure 4C). If NU9056 action was mediated through inhibiting Tip60 enzymatic activity, inhibition of cell proliferation by NU9056 would be expected. Indeed, proliferation was found to be reduced in the presence of NU9056 in all CaP cell lines tested as measured by sulforhodamine B (SRB) assay (Table 2, Supplementary Figure S3, S4). Interestingly LNCaP cells, which are androgen responsive and express a mutated but functional AR as well as wild type p53, and the bone metastasis derived PC3 cells, which do not express a functional AR and are p53 null, displayed similar GI_50_ concentrations (24 µM ±2 and 27 µM ±2 for LNCaP and PC3 cells, respectively). However, LNCaP-AI cells, a sub-line of LNCaP cells serially maintained in steroid-depleted media, which still express a functional AR and are a model of castrate resistant CaP post androgen ablation therapy, have a lower GI_50_ (24 µM ±2 and 16 µM ±1 for LNCaP and LNCaP-AI, respectively). Other cell line models of androgen independence, e.g. LNCaP-CdxR, which is serially maintained in the presence of bicalutamide and CWR22rv1, show significantly greater sensitivity to NU9056 than the parental LNCaP cell line (GI_50_ values of 12 µM ±2.5 and 7.5 µM ±0.2 (p<0.05 and p<0.0005, respectively)). Tip60 levels were measured by Western blotting in these cell lines (Figure 4D) to reveal that the most sensitive cell line, CWR22rv1, actually expresses the most Tip60.

To test whether NU9056 can reduce the survival of CaP cells, colony forming ability was evaluated following exposure of LNCaP cells to 24 µM (GI_50_) NU9056 for 24 hours. Colony forming ability was reduced following NU9056 exposure, which was statistically significant (p<0.05) (Figure 4E).

### NU9056 Treatment Causes Apoptosis via Caspase Activation

**Figure 5:**
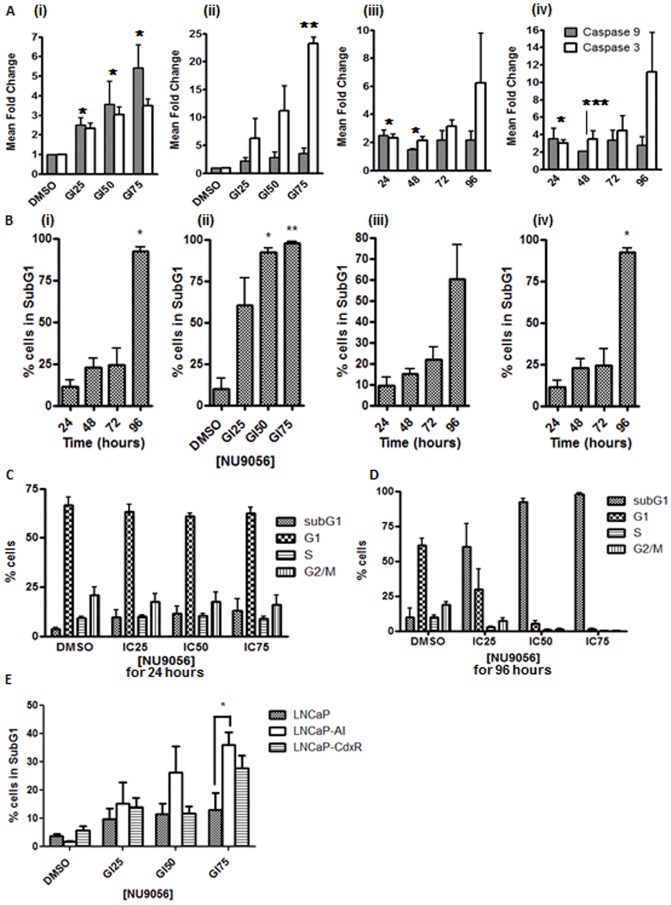
NU9056 reduces LNCaP cell survival by inducing apoptosis. (A) LNCaP cells were seeded onto 6 well plates for 24 hours, then increasing doses of NU9056 were applied for (i) 24 hours, (ii) 96 hours or (iii) GI_25_ (17 µM) or (iv) GI_50_ (24 µM) was applied over 4 days. All cells were collected and fixed with cytofix/cytoperm (BD) then caspase 3 and caspase 9 assay kits (BD) were used to assess their activity by flow cytometry. Fluorescence was detected on the FL-1 channel of the FACSCAN. (B) Analysis of the SubG1 population was performed on these same cells using propidium iodide to stain cellular DNA. LNCaP cells were seeded onto 6 well plates for 24 hours, then NU9056 was applied for (C) 1 or (D) 4 days. (E) LNCaP, LNCaP-AI and LNCaP-CdxR cells were seeded out onto 6 well plates and NU9056 was applied for 24 hours. Analysis of SubG1 was performed as described above. All cells were collected and fixed with cytofix/cytoperm (BD) then cell cycle analysis was performed using propidium iodide to stain cellular DNA. All FACS data was analysed using WinMDI. All experiments were performed 3 times and the mean ± standard error is shown. *p-value <0.05; **p-value <0.005; ***p-value <0.001.

The effects of NU9056 on cell cycle phase distribution and apoptosis induction were also tested in LNCaP cells. To assess apoptotic effects, FACS analysis for activated caspase 3 and activated caspase 9 was used. NU9056 resulted in both caspase 3 and caspase 9 activation in a time- and concentration-dependent manner (Figure 5A, Supplementary Figures S5 and S6). The levels of apoptosis were also compared with other HAT inhibitors demonstrating that NU9056, with its greater specificity for Tip60, can induce apoptosis at similar levels to the more promiscuous inhibitors (Supplementary Figure S7). Analysis of the sub-G1 population in LNCaP cells (Figure 5B) confirmed the induction of apoptosis in a time- and concentration-dependent manner for NU9056. However, under no conditions of exposure to NU9056 did we observe any G1 or G2M cell cycle arrest in LNCaP cells; only accumulation in the sub-G1 phase was seen (Figure 5C, D). To determine whether castrate resistant cell lines are more sensitive to NU9056 as suggested by GI_50_ determination a comparison of LNCaP cells with LNCaP-AI and LNCaP-CdxR cells after treatment with NU9056 for 24 hours was carried out. Indeed, LNCaP-AI and LNCaP-CdxR cells appear to be more sensitive to NU9056 than LNCaP cells as shown by a greater population being in the Sub-G1 phase of the cell cycle (Figure 5E). Upon using the LNCaP GI_75_ dose (36 µM) a significant increase in Sub-G1 was seen in LNCaP-AI cells (p = 0.036) compared to LNCaP cells. Similar trends were seen for LNCaP-CdxR although this was not statistically significant (p = 0.1679).

### NU9056 Treatment Reduces PSA Expression in LNCaP Cells

**Figure 6:**
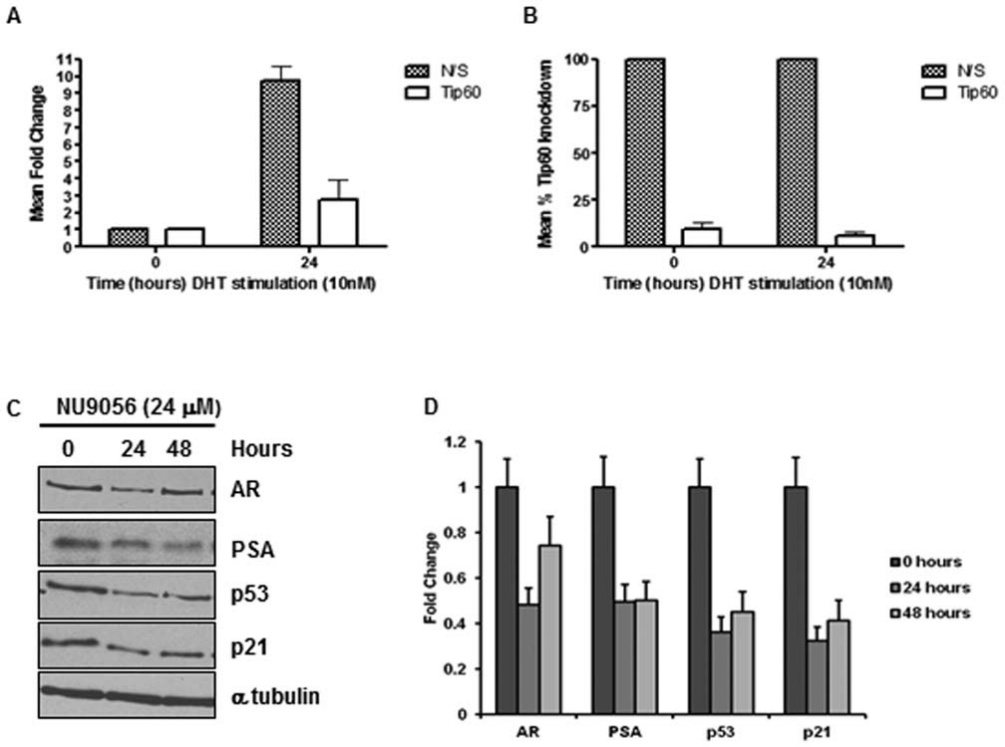
NU9056 reduces PSA and p53 protein levels. To confirm the effects of Tip60 on androgen receptor activity we used 2.5 nM siRNA specifically targeted against Tip60 in LNCaP cells, or non-silencing control. Knockdown was achieved after 48 hours in steroid depleted medium after which time 10 nM DHT was applied to induce androgen receptor activity and PSA expression. RNA was collected after 24 hours DHT stimulation, reverse transcription and real-time PCR performed. Expression of (A) PSA and (B) Tip60 was normalised relative to HPRT1 expression. (C) LNCaP cells were treated with 24 µM NU9056 over 48 hours and protein samples were collected in SDS sample buffer. Protein analysis was carried out by SDS PAGE and Western blotting for p53, p21, AR, PSA and alpha tubulin. (D) Densitometry was performed on Western blots. All experiments were performed twice and the mean ± standard deviation is shown.

Tip60 has been reported as an AR co-activator [31], which plays a role in CaP development. To confirm the role of Tip60 in PSA expression, siRNA was used to knockdown Tip60 levels in LNCaP cells and PSA mRNA levels monitored in response to androgen (dihydrotestosterone (DHT)) stimulation. In the presence of a non-silencing siRNA PSA was induced by approximately 10-fold in response to DHT (Figure 6A). However, after knockdown of Tip60 (Figure 6B) only a 2.5 fold increase was observed (Figure 6A). To investigate the effects of NU9056 on AR function, LNCaP cells were treated with 24 µM NU9056 over a 48 hour period, whereupon levels of AR and PSA protein were assessed by Western blotting. In addition, we investigated the levels of p53 and its target gene p21, as p53 is also a target for Tip60. We discovered that the levels of both AR and p53 were reduced after 24 hours by 2- and 3-fold respectively, and that the products of the target genes, p21 and PSA, were also reduced by 3- and 2-fold respectively (Figure 6C,D). This result suggests that indeed Tip60 is involved in AR and p53 signalling in this cell line and that NU9056 may, by modulating Tip60 acetylation activity, affect these important downstream targets.

### NU9056 Inhibits the DNA Damage Response in Prostate Cancer Cells

**Figure 7:**
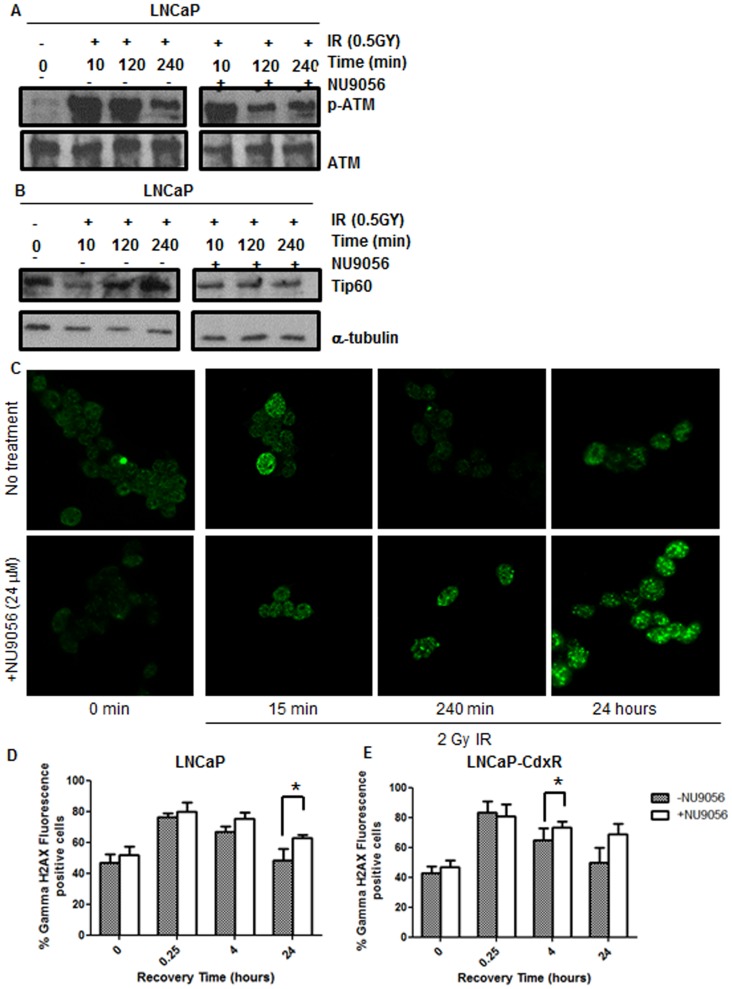
NU9056 inhibits Tip60 accumulation and ATM phosphorylation in response to ionising radiation. To demonstrate the inhibition of Tip60 activity by NU9056, levels of pATM, Tip60 and γH2AX were investigated in response to IR in the presence and absence of drug. LNCaP cells were treated with 24 µM NU9056 or vehicle control for 1 hour prior to 0.5 Gy IR. Protein lysates were collected at various time points post-IR and analysed by Western blotting for (A) pATM and (B) Tip60 levels. (C) 293T, (D) LNCaP and (E) LNCaP-CdxR cells were pretreated with NU9056 (24 µM) or vehicle control for 1 hour prior to 2 Gy IR. Cells were fixed and stained for γH2AX foci over time and foci determined by immunofluorescence for 293T cells and flow cytometry for LNCaP and LNCaP-CdxR. Experiments were repeated 3 times with representative images shown and quantified data shown as mean % cells stained for γH2AX ± standard deviation.

Tip60 is known to play a role in the DNA damage response [25]. Upon exposure to IR Tip60 is activated, which results in acetylation of histone proteins and activation of ATM and p53 [4]. To test whether NU9056 could inhibit the DNA damage response via inhibition of Tip60 acetylase activity, the activation of ATM was assessed by Western blotting in response to IR after pre-treatment with NU9056. In response to IR, pATM levels were increased dramatically within 10 minutes. Over time pATM levels gradually fell. However, in the presence of NU9056 this decrease was much faster (Figure 7A). Furthermore, in response to IR levels of Tip60 protein were stabilised resulting in accumulation. Cells which were pre-treated with NU9056 did not demonstrate Tip60 accumulation (Figure 7B), which may explain higher turnover of pATM levels.

Tip60 acetylase activity is required to facilitate the ubiquitin ligase UBC13-mediated ubiquitination and subsequent destruction of γH2AX [32]. Therefore, inhibition of Tip60 acetylase activity will prevent the down-regulation of the γH2AX signal. To test this, 293T cells were treated with NU9056 (24 µM) or vehicle control for 1 hour prior to IR (2 Gy) exposure. The cells were then fixed and γH2AX foci visualised by immunofluorescence. Compared to vehicle control, γH2AX formation was still clearly enriched as much as 24 hours after IR stimulation (Figure 7C) suggesting that the repair of DNA damage is impaired. This is also seen in LNCaP and LNCaP-CdxR cells which were assessed by flow cytometry for γH2AX. In LNCaP cells, the number of foci were significantly higher after 24 hours recovery in the presence of inhibitor (p = 0.0277) (Figure 7D) whilst in LNCaP-CdxR cells a significant difference is seen after 4 hours recovery (p = 0.0324) (Figure 7E). An increase in γH2AX is also seen at 24 hours recovery but was found not to be statistically significant.

## Discussion

Protein acetylation, as a regulatory mechanism, is proving to be important in many cellular pathways, not just gene transcription via histone modification. Both sets of enzymes responsible for regulating acetylation, HATs and HDACs, are de-regulated in disease states. Therefore, targeting both types of enzymes with small molecule inhibitors as a therapeutic strategy is valid. Inhibitors against HDACs have been found to be successful in clinical trials; however, HAT inhibitors are at an earlier stage of development. Recently, there have been some putative HAT inhibitors described, although none appear able to distinguish significantly between the different HAT family members and none have been specifically developed against Tip60, a HAT enzyme which appears to play a particular role in CaP development and progression. To address this point, we identified a HAT inhibitor, using HTS and targeted compound synthesis, which inhibits Tip60 over other HAT enzymes.

The requirement to fully validate HTS hits through resynthesis is widely accepted as material in commercial compound collections may include unidentified impurities, or may degrade on storage, typically as frozen DMSO solutions, giving false positives. In this case, a literature synthesis for **1** was not available and a route had to be developed. The first scheme attempted (Part 1) did not give the target compounds, **1**, or its desmethyl analogue; however, the isocyanato and disulfide analogues **4–7** were prepared. Compound **1** was prepared successfully via an alternative route (Part 2).

The biological activity observed for the disulfides **5** and **7** (NU9056), prompted us to investigate the activity of other simple aromatic and heteroaromatic disulfides. Interestingly, these compounds were devoid of Tip60 inhibitory activity, indicating that Tip60 inhibition is not solely due to the presence of the disulfide group. Similarly, the bromothiophene analogue of isothiazole **1** was inactive.

Isothiazolones have been previously reported to target the acetylase activity of many HAT enzymes including p300 and PCAF [24]. However, a specific inhibitor for Tip60 has not been described. There are many benefits to be gained by targeting this protein due to the diverse cellular processes in which Tip60 is implicated. For example, not only does this protein function to increase the transcriptional activity of AR and p53, but it can also play a role in DNA repair where it can acetylate histone proteins to mark sites of DNA damage and activate ATM [25].

In this report, we have prepared an isothiazolone compound, NU9056 (**7**) that targets Tip60 HAT activity selectively resulting in reduced acetylation of histone proteins *in vitro*. Tip60 has been found to be aberrantly expressed in a number of cancers, including prostate and skin cancers. Specifically, Tip60 can acetylate the AR, a key transcription factor in CaP, to promote increased AR transcriptional activity [6] and Tip60 expression has also been shown to correlate with disease progression [14]. Thus, targeting the acetylase activity of this protein could be beneficial to patients suffering with castrate resistant CaP that no longer responds to androgen deprivation therapy. Therefore, to test the ability of NU9056 to inhibit HAT activity in cells we have used CaP cell line models. In these cell lines we have demonstrated the inhibitory effect of NU9056 against the HAT activity of Tip60. Furthermore, acetylation of non-histone proteins such as tubulin was found to be reduced in these cell lines in response to NU9056. Interestingly, the levels of AR and p53 decreased slightly after NU9056 treatment in LNCaP cells supporting previous reports that acetylation enhances the stability of these proteins [33], [34]. Due to the importance of AR and p53 and their presence in LNCaP cells, this may explain why both apoptosis is increased and proliferation is decreased in response to NU9056 in a concentration and time dependent manner. Nevertheless, there are differences in the sensitivity between different CaP cell lines which cannot be explained solely by AR or p53 status. This latter observation suggests that the activity of Tip60 towards AR and p53 is not a contributing factor to the cellular response to the drug. However in response to DNA damaging IR, which activates the acetylase activity of Tip60, we find that NU9056 inhibits the development of the pATM signal and impedes the stabilisation of Tip60 itself, potentially via inhibition of auto-acetylation. Furthermore, NU9056 impairs cell survival in response to IR and impairs the removal of the γH2AX mark potentially hindering DNA repair.

Interestingly, cell line models of castrate resistance appear to be more sensitive to NU9056. Increased levels and stability of Tip60 in androgen-insensitive cells, due to chronic growth in androgen ablated conditions, has been reported in other similar cell line models [35], human CaP xenografts and biopsy samples from castrate resistant patients [14]. It is possible that androgen-insensitive cells are more dependent on Tip60 levels for their growth, compared to their androgen responsive counterparts, resulting in greater sensitivity to NU9056. Indeed, initial studies show that the levels of Tip60 protein vary between the cell lines tested, with the more NU9056 sensitive cell lines, CWR22rv1 and LNCaP-CdxR demonstrating the highest levels of Tip60. Differences in enzymatic activity of Tip60 between cell lines may also be important. This hypothesis should be fully tested in future studies in a number of cell lines and *in vivo* model systems to conclusively determine the mechanism of sensitivity. As to whether NU9056 is generally toxic to the cells; we believe that this is unlikely for the most part. Firstly, there was no change in γH2AX staining when NU9056 was applied to cells suggesting no induction of DNA damage. Secondly differential effects were seen depending on the cell line suggesting generally toxicity was not the cause of detrimental cellular effects. However, at higher doses a role for general toxicity may become apparent.

Overall, a therapeutic role for Tip60 inhibitors in the treatment of castrate resistant CaP is supported by the chemical biology and molecular genetic studies described in this paper.

## Materials and Methods

### Reagents and Antibodies

NU9056, 1,2-bis(4-pyridyl)-ethane (Sigma) and related compounds were dissolved in DMSO as 10 mmol/L stocks and stored at −20°C. Anti-histone H4-acetyl lysine 8 (ab15823), acetyl lysine 16 (ab61240) and Anti-histone H3-acetyl lysine 14 (ab61232) antibodies were obtained from AbCam (Cambridge, UK). Anti-Tip60 (07-038) antibody was obtained from Millipore (MA, USA). Anti-androgen receptor (G122-77) antibody was obtained from BD (NJ, USA). Anti-PSA (C-19; sc7638) antibody was obtained from Santa Cruz Biothechnologies (USA). Anti-alpha tubulin clone DM1A (T9026), anti-acetylated tubulin clone 6-11B-1 (T7451) antibodies were obtained from Sigma (MO, USA). Anti- p53 (Do-1) and anti-p21 (Ab-4) antibodies were obtained from Calbiochem (Germany).

### Cell Culture

Tissue culture reagents were purchased from Sigma. LNCaP, CWR22Rv1 and PC3 cells, obtained from the American Type Culture Collection (Manassas, VA, USA), were maintained in RPMI 1640 media supplemented with 10% (v/v) fetal calf serum (FCS), and 2 mM L-glutamine at 37°C in 5% CO_2_ atmosphere. LNCaP-AI and LNCaP-cdxR cells were generated and maintained as previously described [14], [36].

### Synthesis of HAT Inhibitors

This is described in detail in supplementary materials and methods.

### *In vitro* Histone Acetyl-transferase Assay

HAT assays were performed as previously described [37]. Briefly, HAT assay buffer (50 mM Tris pH 8, 0.5 mM EDTA pH 8, 10% glycerol), histones (Sigma) and recombinant HAT enzymes (produced and purified as described in supplementary materials and methods) were combined with putative HAT inhibitor molecules and incubated at room temperature for 10 mins. ^3^H acetyl-CoA was added and incubated at 30°C for 30 minutes. Samples were blotted onto filter paper, washed and dried. Scintillation counts were detected using a Wallac 1450 Microbeta Trilux liquid scintillation and a luminescence counter. Assays were performed in quadruplicate.

### Western Blotting

All cell lysates for Western analyses were collected in SDS loading buffer (0.125 M Tris pH 6.8, 2% SDS, 10% (v/v) glycerol, 10% (v/v) β-mercaptoethanol, and 0.01% (w/v) bromophenol blue). Samples were separated by SDS-PAGE prior to transfer to nitrocellulose membrane (Hybond-C, Amersham). Membranes were blocked with 5% (w/v) Marvel™ at room temperature for 1 hour, incubated with primary antibody overnight at 4°C followed by incubation with the appropriate horseradish peroxidise conjugated secondary antibody (Dako). Proteins of interest were visualised with ECL reagent (Amersham, UK).

### Sulforhodamine B Growth Inhibition Assays

Cells were seeded into 96-well plates at a density appropriate for exponential growth at the beginning of the assay. After overnight attachment, cells were treated with NU9056 over a range of concentrations for 3 days (equivalent to approximately 3 population doubling times). Cells were then fixed in 10% (w/v) TCA and stained with sulforhodamine B (SRB) as previously described [38]. The concentrations required to inhibit cell growth by 50% (GI_50_) were calculated using GraphPad Prism (San Diego, CA, USA) software.

### Colony Forming Assay

LNCaP cells were seeded out onto 90 mm dishes, exposed to increasing amounts of IR, then re-seeded out at varying cell densities and incubated for 14 days to allow colony formation. Medium was then removed and cells gently washed and stained with crystal violet. Colonies were counted and the surviving fraction calculated.

### FACS Analysis

LNCaP cells were seeded onto 6 well plates and allowed to attach overnight. The appropriate concentration of NU9056 was then applied for the indicated time. Medium containing any floating cells was removed and retained, and cells were trypsinised from the plates and added to the removed media. Cells were fixed using 50 µl cytofix/cytoperm (BD) then caspase 3 and caspase 9 assay kits (BD) were used to detect caspase activity, according to the manufacturer’s protocol. Fluorescence was detected on the FL-1 channel of FACSCAN. Cell cycle profiles were generated by propidium iodide staining for 10 minutes in the presence of RNase and 5% (v/v) Triton.

### γH2AX Immunofluorescence

293T cells were seeded onto coverslips in 6 well plates and allowed to adhere for 24 hours. NU9056 (24 µM) or DMSO was applied to the cells for 1 hour prior to exposure to 2 Gy IR. Cells were then fixed in 4% (v/v) paraformaldehyde and stained for γH2AX as previously described [39] using primary anti-phospho-histone H2AX (Ser139) antibody (clone JBW301, mouse monoclonal antibody; Upstate, Millipore Corp).

### γH2AX FACS Analysis

LNCaP cells were seeded onto 6 well plates and allowed to attach overnight. The appropriate concentration of NU9056 was then applied for the indicated time. Medium containing any floating cells was removed and retained, and cells were trypsinised from the plates and added to the removed media. Cells were fixed using 50 µl cytofix/cytoperm (BD) then incubated in primary anti-phospho-histone H2AX (Ser139) antibody for 2 hours. Cells were then washed twice in 1 X Permwash (BD) and incubated in secondary rabbit anti-mouse FITC conjugated antibody (DAKO) for 2 hours in the dark. Fluorescence was detected on the FL-1 channel of FACSCalibur (BD).

## Supporting Information

Figure S1 **Inhibition of** ***in vitro*** **HAT activity.** *In vitro* HAT assays were performed using histone proteins, recombinant HAT enzymes and ^3^H acetyl CoA in the presence and absence of HAT inhibitors. Scintillation counts were detected and % inhibition calculated. Experiments were performed in quadruplicate and repeated 3 times. Mean % inhibition was calculated ± standard error and used to determine IC_50_ values for each putative HAT inhibitor.
(TIF)

Figure S2 **Densitometry of Western blots.** Densitometry was performed using QuantityOne (BioRAD) on Westerns shown in Figure 2. All data is normalised to background and loading controls then expressed as fold change compared to DMSO controls ± standard deviation.
(TIF)

Figure S3 **IC50 determination from LNCaP growth curves.** LNCaP cells were seeded out onto 96 well plates and incubated in the presence of HAT inhibitor for 3 doubling times. Cells were fixed and sulforhodamine B (SRB)assays performed. Individual experimental repeats are shown and the corresponding IC_50_ values.
(TIF)

Figure S4 **NU9056 inhibits growth in prostate cancer cell lines.** Prostate cancer cells were seeded out onto 96 well plates and incubated in increasing concentrations of NU9056 for 3 doubling times. Cells were then fixed and sulforhodamine B (SRB) assays performed. Experiments were performed as 6 replicates, repeated on 3 independent occasions. Mean % growth inhibition ± standard deviation is shown.
(TIF)

Figure S5 **Caspase 9 cleavage in LNCaP cells.** LNCaP cells were seeded onto 6 well plates for 24 hours, then NU9056 was applied for 1–4 days. All cells were collected and fixed with cytofix/cytoperm (BD) then caspase 9 assay kit (BD) was used to assess caspase cleavage activity by flow cytometry. Fluorescence was detected on the FL-1 channel of FACSCAN. Experiments were repeated 3 times. Data shown are dot plots of 10,000 events for 1 representative experiment.
(TIF)

Figure S6 **Caspase 3 cleavage in LNCaP cells.** LNCaP cells were seeded onto 6 well plates for 24 hours, then NU9056 was applied for 1–4 days. All cells were collected and fixed with cytofix/cytoperm (BD) then caspase 3 assay kit (BD) was used to assess caspase cleavage activity by flow cytometry. Fluorescence was detected on the FL-1 channel of FACSCAN. Experiments were repeated 3 times. Data shown are dot plots of 10,000 events for 1 representative experiment.
(TIF)

Figure S7 **Caspase 3 cleavage in LNCaP cells in response to HAT inhibition.** LNCaP cells were seeded out onto 6 well plates and incubated with GI50 concentrations of HAT inhibitors for 96 hours. Cells were then harvested and caspase 3 cleavage detected using an anti-cleaved caspase 3 FITC conjugated antibody and flow cytometry. (A) Mean fold change of 3 experiments ± standard deviation is shown. (B) Dot plots of 10,000 events are shown for 1 representative experiment. Docetaxol was included as a positive control to induce apoptosis.
(TIF)

Supplementary Information S1 Additional materials,methods, results and references. (DOCX)
