# Supplementary figures and images for: Characterisation of a Tip60 Specific Inhibitor, NU9056, in Prostate Cancer
Source: PLoS One. 2012 Oct 8;7(10):e45539. doi: 10.1371/journal.pone.0045539 (PMC3466219; doi:10.1371/journal.pone.0045539)

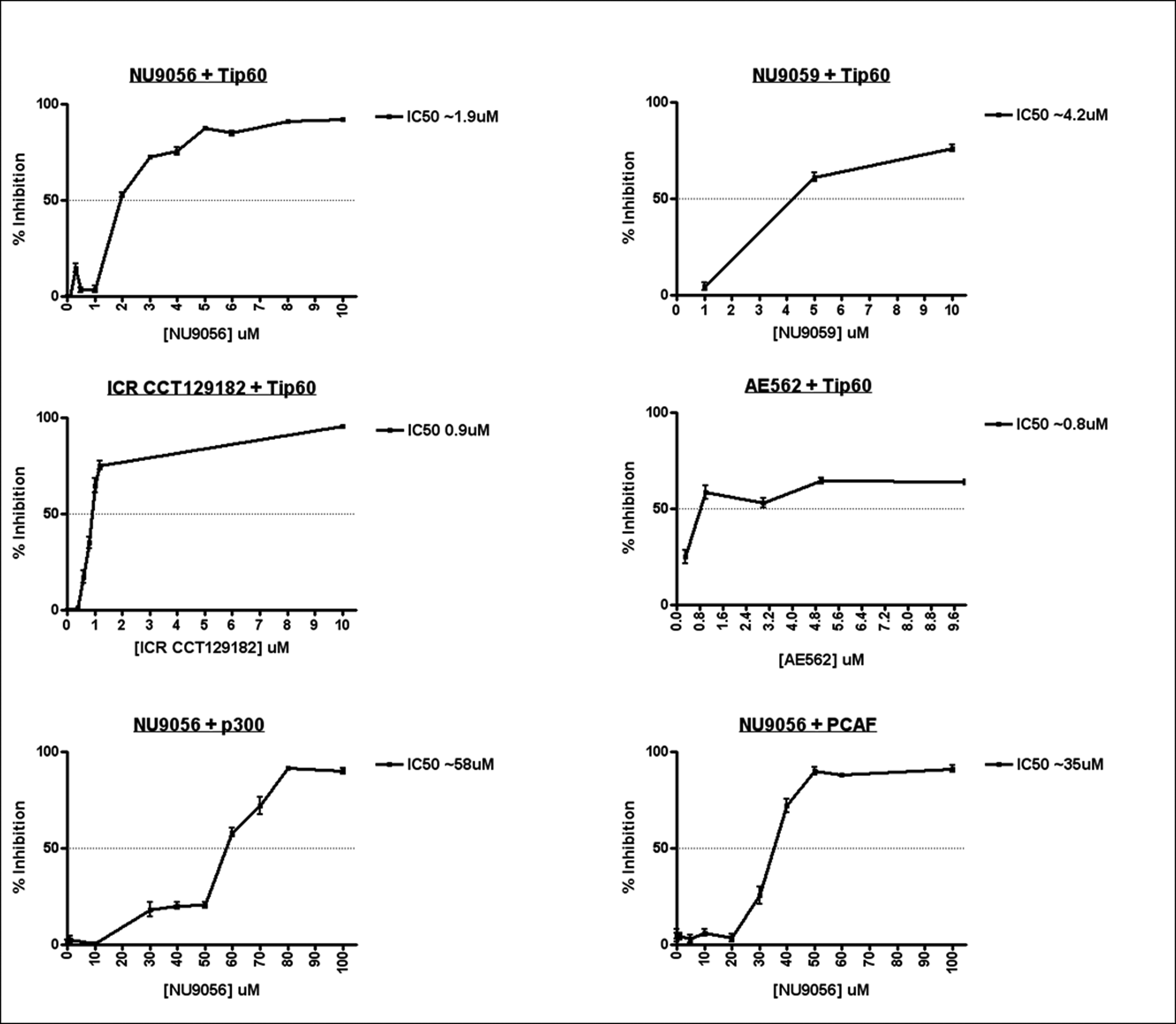

Supplement: Figure S1 — Inhibition of in vitro HAT activity. In vitro HAT assays were performed using histone proteins, recombinant HAT enzymes and 3H acetyl CoA in the presence and absence of HAT inhibitors. Scintillation counts were detected and % inhibition calculated. Experiments were performed in quadruplicate and repeated 3 times. Mean % inhibition was calculated ± standard error and used to determine IC50 values for each putative HAT inhibitor. (TIF) [file pone.0045539.s001.tif]

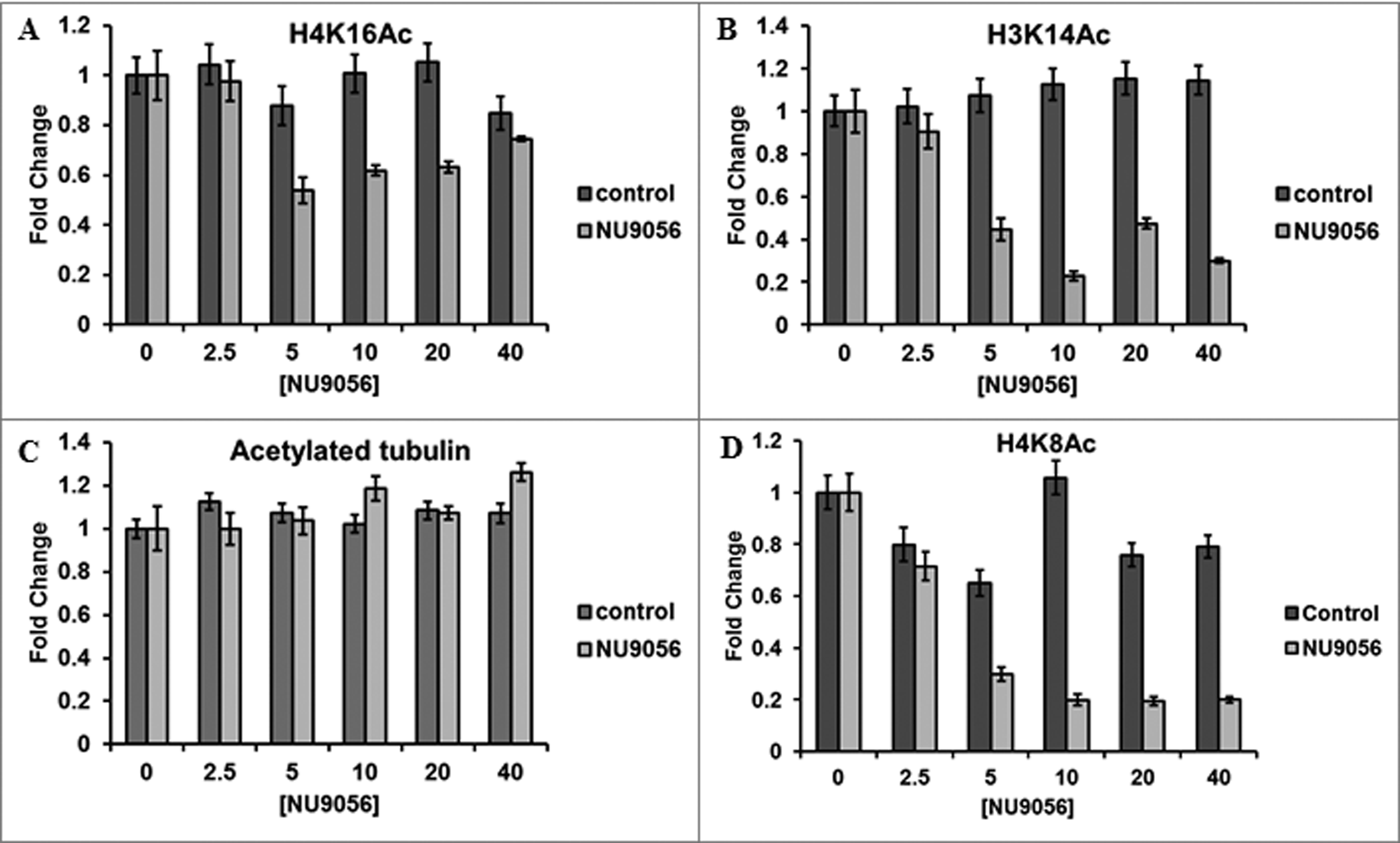

Supplement: Figure S2 — Densitometry of Western blots. Densitometry was performed using QuantityOne (BioRAD) on Westerns shown in Figure 2. All data is normalised to background and loading controls then expressed as fold change compared to DMSO controls ± standard deviation. (TIF) [file pone.0045539.s002.tif]

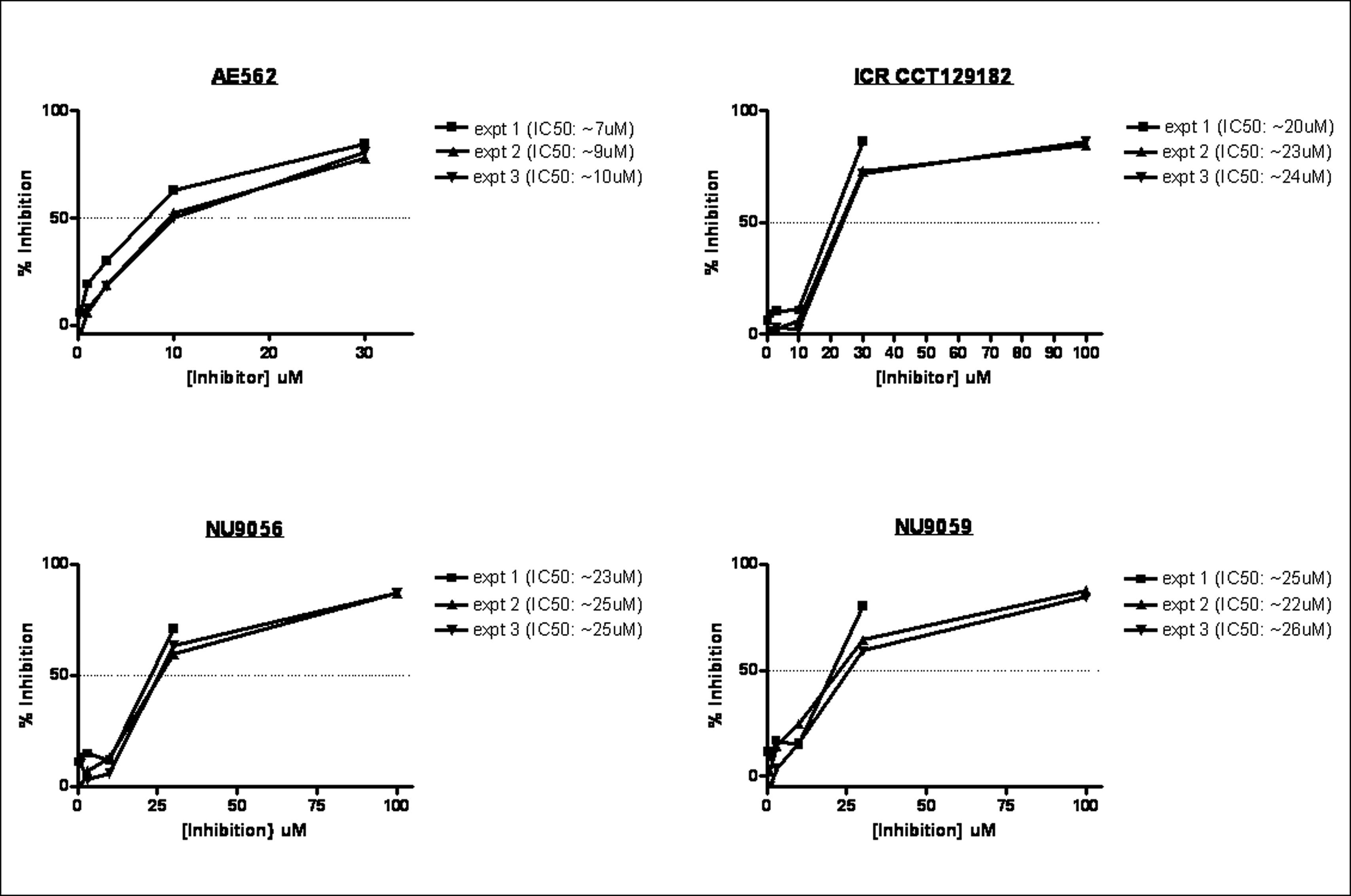

Supplement: Figure S3 — IC50 determination from LNCaP growth curves. LNCaP cells were seeded out onto 96 well plates and incubated in the presence of HAT inhibitor for 3 doubling times. Cells were fixed and sulforhodamine B (SRB)assays performed. Individual experimental repeats are shown and the corresponding IC50 values. (TIF) [file pone.0045539.s003.tif]

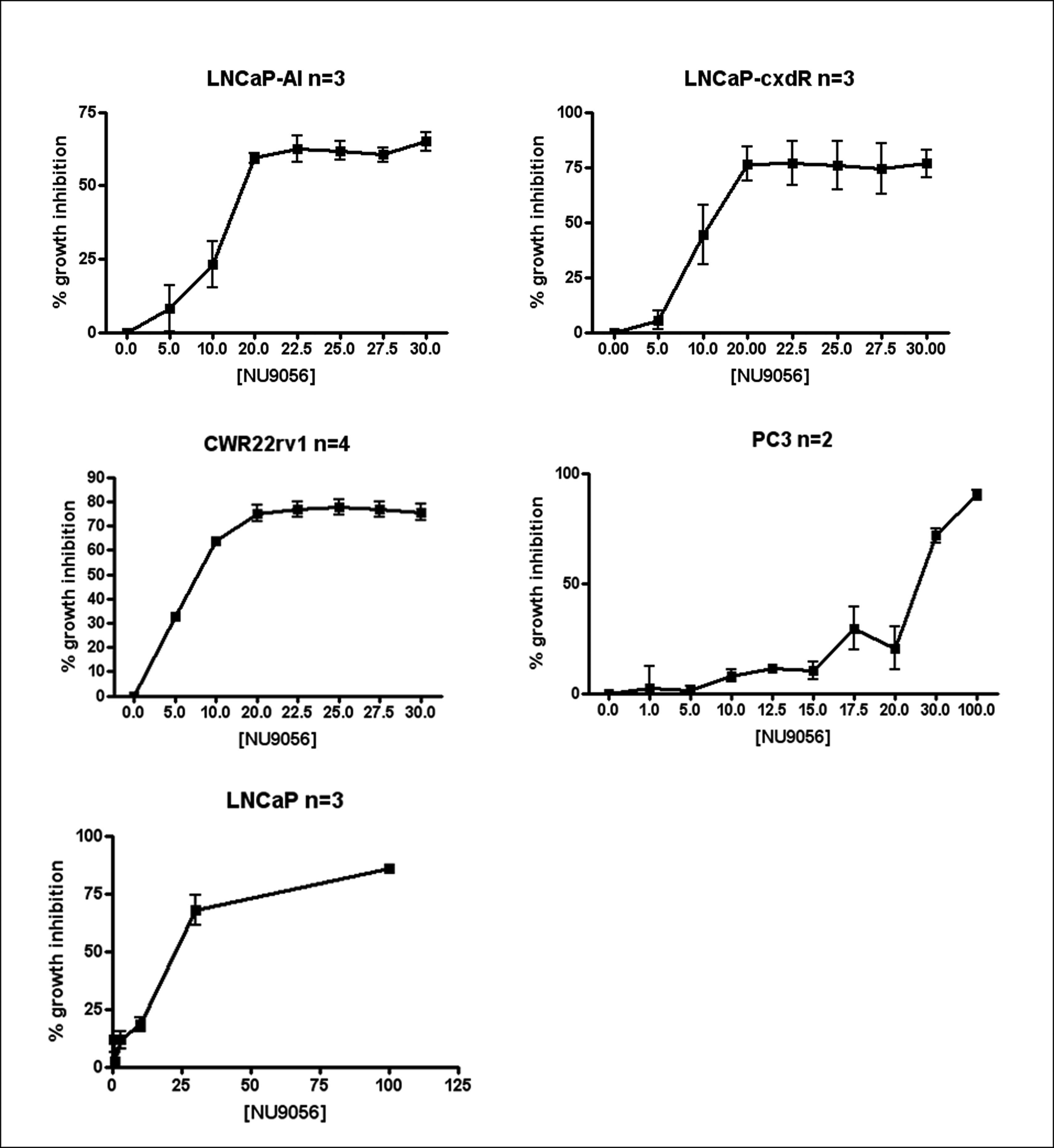

Supplement: Figure S4 — NU9056 inhibits growth in prostate cancer cell lines. Prostate cancer cells were seeded out onto 96 well plates and incubated in increasing concentrations of NU9056 for 3 doubling times. Cells were then fixed and sulforhodamine B (SRB) assays performed. Experiments were performed as 6 replicates, repeated on 3 independent occasions. Mean % growth inhibition ± standard deviation is shown. (TIF) [file pone.0045539.s004.tif]

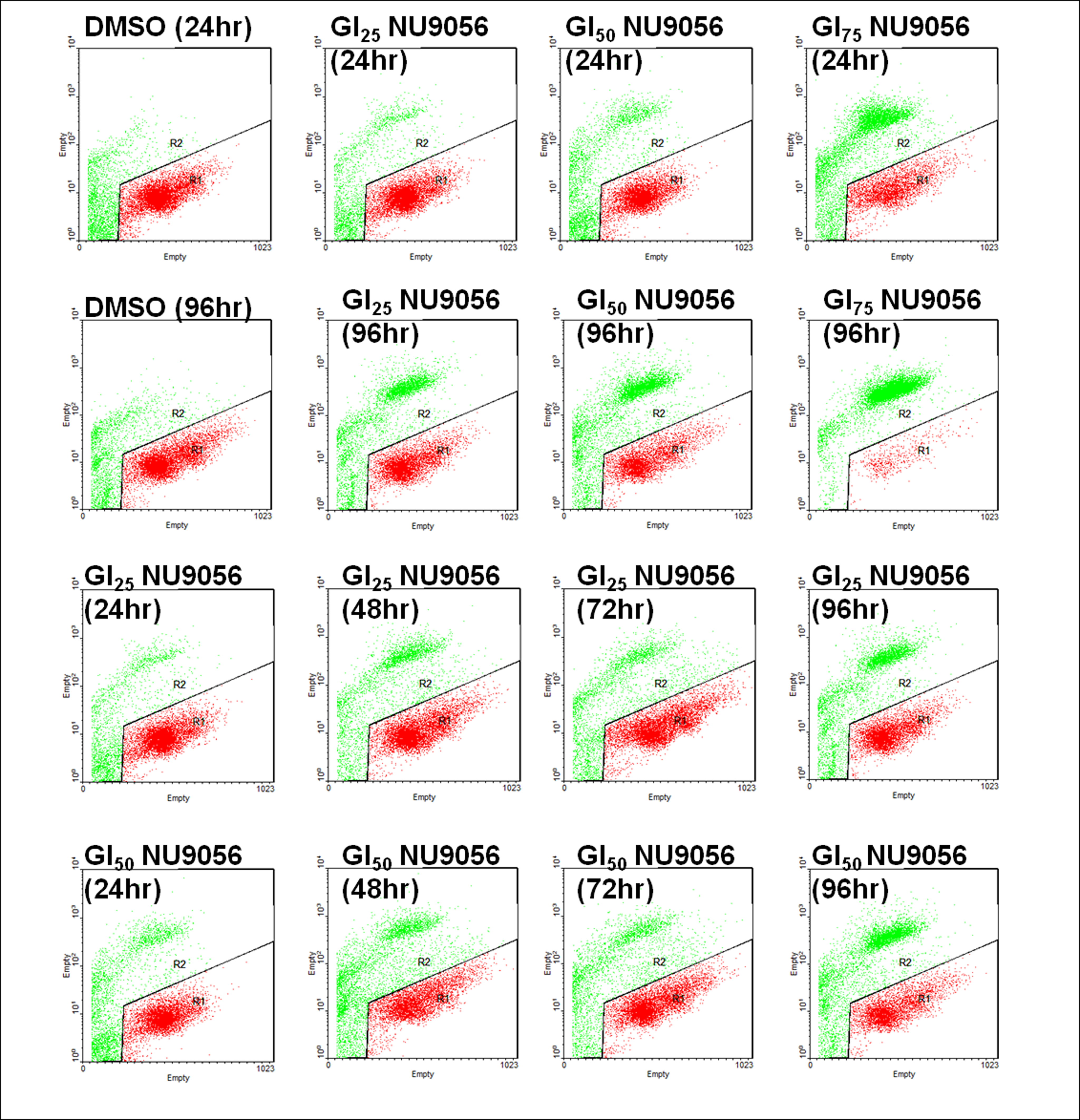

Supplement: Figure S5 — Caspase 9 cleavage in LNCaP cells. LNCaP cells were seeded onto 6 well plates for 24 hours, then NU9056 was applied for 1–4 days. All cells were collected and fixed with cytofix/cytoperm (BD) then caspase 9 assay kit (BD) was used to assess caspase cleavage activity by flow cytometry. Fluorescence was detected on the FL-1 channel of FACSCAN. Experiments were repeated 3 times. Data shown are dot plots of 10,000 events for 1 representative experiment. (TIF) [file pone.0045539.s005.tif]

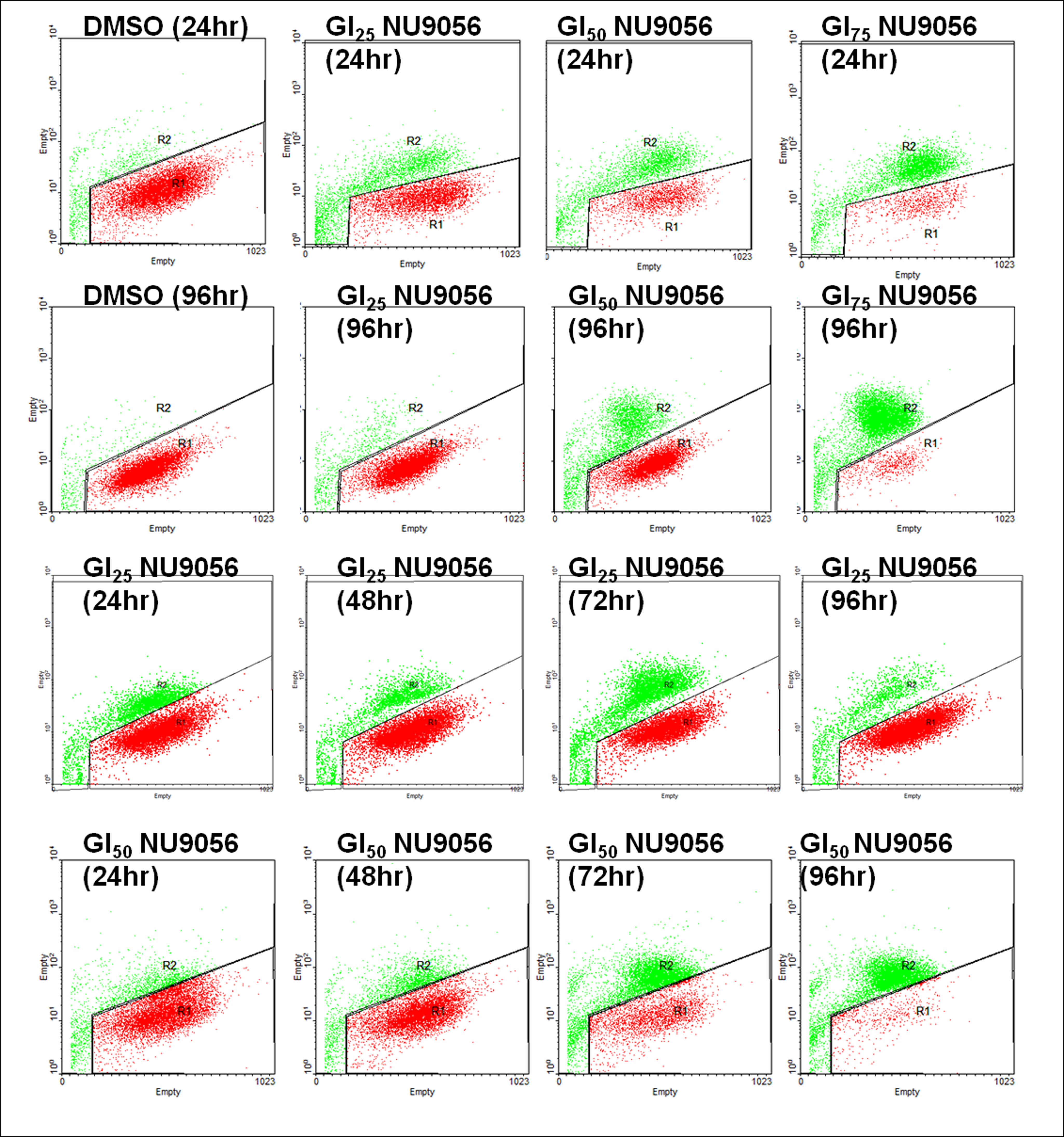

Supplement: Figure S6 — Caspase 3 cleavage in LNCaP cells. LNCaP cells were seeded onto 6 well plates for 24 hours, then NU9056 was applied for 1–4 days. All cells were collected and fixed with cytofix/cytoperm (BD) then caspase 3 assay kit (BD) was used to assess caspase cleavage activity by flow cytometry. Fluorescence was detected on the FL-1 channel of FACSCAN. Experiments were repeated 3 times. Data shown are dot plots of 10,000 events for 1 representative experiment. (TIF) [file pone.0045539.s006.tif]

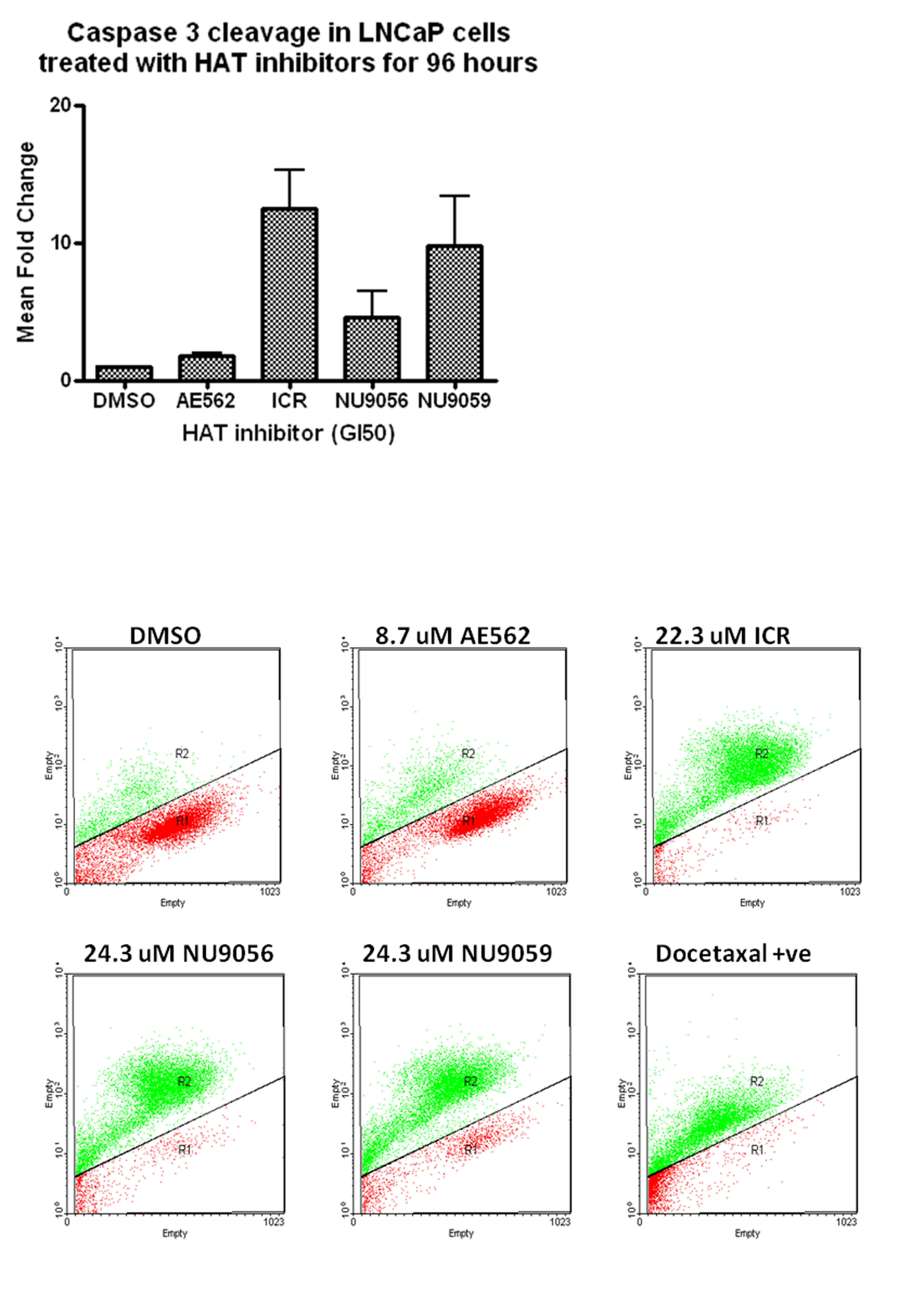

Supplement: Figure S7 — Caspase 3 cleavage in LNCaP cells in response to HAT inhibition. LNCaP cells were seeded out onto 6 well plates and incubated with GI50 concentrations of HAT inhibitors for 96 hours. Cells were then harvested and caspase 3 cleavage detected using an anti-cleaved caspase 3 FITC conjugated antibody and flow cytometry. (A) Mean fold change of 3 experiments ± standard deviation is shown. (B) Dot plots of 10,000 events are shown for 1 representative experiment. Docetaxol was included as a positive control to induce apoptosis. (TIF) [file pone.0045539.s007.tif]
