# Supplementary material for: Characterisation of a Tip60 Specific Inhibitor, NU9056, in Prostate Cancer
Source: PLoS One. 2012 Oct 8;7(10):e45539. doi: 10.1371/journal.pone.0045539 (PMC3466219; doi:10.1371/journal.pone.0045539)
Supplement: Supplementary Information S1 Additional materials,methods, results and references. — (DOCX) [file pone.0045539.s008.docx]

**Supplementary Information**

**Materials and Methods**

***Synthesis of HAT inhibitors****-* 2-Bromo-3-methylthiophene, diphenyl disulphide, 2,2'-dithio(bis)benzothiazole, 2,2'- and 4,4'-dithiodipyridine (Aldrithiols) were purchased from the Aldrich Chemical Company.

**3,3-Dibromo-2-methylacrylaldehyde (3a)**

To pyruvic aldehyde dimethylacetal **2a** (5.13 mL, 42.3 mmol) and triphenylphosphine (20.2 g, 77.0 mmol) suspended in trifluoromethylbenzene (20 mL) was added dropwise a solution (pre-filtered through basic alumina) of carbon tetrabromide (12.8 g, 38.5 mmol) in trifluoromethylbenzene (80 mL). The mixture was heated at 100 ^o^C for 3 h, cooled and filtered to remove triphenylphosphine oxide. The solvent was removed and the crude product was purified by chromatography (silica; elution with 25% dichloromethane in petrol) to afford **2a** as a pale yellow oil (2.23 g, 24 %). ^1^H NMR (CDCl_3_, 300 MHz) δ 1.86 (s, 3H, CH_3_), 9.89 (s, 1H, CHO); ^13^C NMR (CDCl_3_, 75 MHz) δ 17.3, 113.6, 140.5, 189.6.

**4-Methyl-5-thiocyanatoisothiazole (4)**

To 3,3-dibromo-2-methylacrylaldehyde (1.45 g, 6.36 mmol) in dimethylformamide (10 mL) was added ammonium thiocyanate (0.97 g, 12.7 mmol). The resulting solution was heated at 70 ^o^C for 2 h, cooled, diluted with brine and extracted with diethyl ether. The ethereal extract was washed with water, dried (MgSO_4_) and concentrated to give an orange liquid. Chromatography (silica; elution with 12% dichloromethane in petrol) gave compound **4**, which was further purified by recrystallisation: yellow crystals (0.59 g, 60 %) from ethyl acetate-petrol. ^1^H NMR (CDCl_3_, 300 MHz) δ 2.42 (s, 3H, CH_3_), 8.41 (s, 1H, isothiazole CH); ^13^C NMR (CDCl_3_, 75 MHz) δ 12.0, 108.0, 138.2, 140.8, 160.2; HRMS (EI^+^) calculated for C_5_H_4_N_2_S_2_: 155.9816; found: 155.9814.

**1,2-Bis(4-methylisothiazol-5-yl)disulfane ( 5)**

Aqueous ammonia (ca. 40 M, 5 mL) was added to a stirred solution of **4** (0.45 g, 2.88 mmol) in dioxane (5 mL) and water (2.5 mL) and the mixture was heated at 90 ^o^C for 1 h. The solution was cooled to room temperature and the solvent was removed *in vacuo*. The red residue was partitioned between water and dichloromethane (DCM). The organic layer was dried (MgSO_4_), filtered and concentrated *in vacuo*. Chromatography (silica; elution with 6% ethyl acetate in petrol) gave compound **5** (0.14 g, 37 %) as a yellow oil. IR 2925, 2855, 2362, 2338, 1725, 1449, 1366, 1282, 1224, 1122, 992, 881, 788, 664 cm^-1^; ^1^H NMR (CDCl_3_, 300 MHz) δ 2.19 (s, 6H, 2 × CH_3_), 8.30 (s, 2H, 2 × isothiazole CH); ^13^C NMR (CDCl_3_, 75 MHz) δ 11.7 (2 × CH_3_), 138.2 (2 × CH_3_*C*), 153.7 (C-S-S-C), 159.8 (2 × CH=N); HRMS (EI^+^) calculated for C_8_H_8_N_2_S_4_: 259.9570; found: 259.9568.

**5-Thiocyanatoisothiazole (6) and 1,2-Bis(isothiazol-5-yl)disulfane (NU9056, 7)**

3,3-Dibromoacrylaldehyde **3b** was prepared from 2,2-dimethoxyethanal **2b** in the manner described above for **3a**. A mixture of **3b** (0.35 g, 1.63 mmol) and ammonium thiocyanate (0.25 g, 3.26 mmol) in dry dimethylformamide (3 mL) was stirred for 2.5 h at 70 °C and allowed to cool to room temperature. Ethyl acetate (50 mL) was added and the mixture was washed (3×) with brine. The organic phase was dried (MgSO_4_) and concentrated *in vacuo*. Chromatography (silica gel; 30% dichloromethane in petrol followed by 5% methanol in dichloromethane) gave:

**6**, yellow solid (94 mg), mp 43 °C. IR (in CDCl_3_) 2960, 2925, 1714, 1621, 1386, 1269, 1220, 1057, 1149, 996, 907, 663 cm^-1^; ^1^H NMR (300 MHz, CDCl_3_) δ 7.20 (d, 1H, *J* 1.5 Hz, CH=CS), 8.36 (d, 1H, *J* 1.5 Hz, CH=N). ^13^C NMR (75 MHz, CDCl_3_) δ 126.4 (*C*H=CS), 158.3 (CH=N), 160.8 (CH=*C*S); HRMS (EI^+^) calculated for C_3_H_2_NS_2_ [M – CN]^+^ 115.9629; found: 115.9629.

**7**, yellow oil (68 mg). IR (in CDCl_3_) 2965, 2932, 2164, 1714, 1559, 1388, 1257, 1227, 1107, 1059, 1007, 820, 725, 664 cm-^1^; ^1^H NMR (300 MHz, CDCl_3_) δ 7.40 (d, 2H, *J* 1.7 Hz, 2 × CH=CS), 8.46 (d, 2H, *J* 1.7 Hz, 2 × CH=N); ^13^C NMR (75 MHz, CDCl_3_) δ 107.8 (2 × SCN), 129.1 (2 × *C*H=CS), 145.2 (2 × CH=*C*S), 158.5 (2 × CH=N); HRMS (EI^+^) calculated for C_6_H_4_N_2_S_4_: 231.9252; found, 231.9255.

**4-Methylisothiazole** **(11)**

Ammonium thiocyanate (3.37 g, 44 mmol) was added to a stirred solution of a mixture of (*E*)- and (*Z*)-3-bromo-2-methylacrylaldehyde **10** (prepared from methacrolein **8** *via* **9**: cf. ref. 40) (2.2 g, 15 mmol) in dimethylformamide (10 mL). The mixture was heated at 70 ^o^C for 16 h, cooled to room temperature, diluted with brine, and extracted with diethyl ether. The combined organic extracts were washed with water (4 × 10 mL), dried (MgSO_4_) and concentrated *in vacuo*. Chromatography (silica; elution with 50 % DCM in petrol) gave compound **11** as a pale yellow oil (0.56 g, 38 %). IR (in CDCl_3_) 3092, 2961, 2926, 2872, 1451, 1363, 1332, 1229, 964, 883, 853, 781 cm^-1^; ^1^H NMR (CDCl_3_, 300 MHz) δ 2.35 (s, 3H, CH_3_), 8.19 (s, 1H, CH=N), 8.27 (s, 1H, CHS); ^13^C NMR (CDCl_3_, 75 MHz) δ 11.59 (CH_3_), 133.9 (CH_3_*C*), 143.1 (=CHS), 158.5 (CH=N); HRMS (EI^+^) calculated for C_4_H_5_NS 99.0143; found 99.0147.

**4-Methyl-5-bromoisothiazole (1)**

To a solution of **11** (0.25 g, 2.5 mmol) in THF (10 mL) cooled to -78 ^o^C was added n-butyl lithium (2.5 M in hexanes, 1.1 mL, 2.7 mmol). After stirring for 15 min, bromine (0.14 mL, 2.8 mmol) was added and the mixture was allowed to warm slowly to room temperature. Aqueous NH_4_Cl was added and the mixture was extracted with diethyl ether, dried (MgSO_4_) and concentrated *in vacuo*. Chromatography (silica; elution with 25 % DCM in petrol) gave compound **1** as a yellow oil (0.043 g, 10 %). IR (in CDCl_3_) 3032, 2960, 2927, 2867, 1537, 1449, 1365, 1323, 1225, 1096, 1036, 941, 880, 780 cm^-1^; ^1^H NMR (CDCl_3_, 300 MHz) δ 2.25 (s, 3H, CH_3_), 8.20 (s, 1H, CH=N); ^13^C NMR (CDCl_3_, 75 MHz) δ 12.1 (CH_3_), 133.2 (CH_3_*C*), 135.2 (=CBr-S), 159.1 (*CH=N*); HRMS (EI^+^) calculated for C_4_H_4_^79^BrNS: 176.9248; found: 176.9249.

***Baculovirus production of His-Tip60***- Wild-type Tip60-His baculovirus was generated in insect cells using the Bac-to-Bac expression system (Invitrogen) according to the manufacturer’s protocol. N-terminal His-tagged wild-type Tip60 was produced by infection of insect cells. Cell lysates were harvested in lysis buffer (50 mM NaH_2_PO_4_, 300 mM NaCl, 10 mM imidazole, pH 8) and applied to Ni-NTA superflow (Qiagen) columns. The columns were washed (50 mM NaH_2_PO_4_, 300 mM NaCl, 20 mM imidazole, pH 8) and Tip60-His was eluted in elution buffer (50 mM NaH_2_PO_4_, 300 mM NaCl, 250 mM imidazole, pH 8). The activity of the protein was confirmed using *in vitro* histone acetylation assays.

***Purification of recombinant HAT enzymes (GST-tagged)****-* Expression constructs, pGEX-p300 and pGEX-PCAF, obtained from Dr Andrew Bannister (Cambridge Cancer Centre, UK), were transformed into XA90 *Escherichia coli* cells. A single colony was selected and grown to an OD 0.5 IPTG (1 mM) induction was carried out for 4 hours then cell pellets were collected, lysed, and sonicated. Supernatants were then passed through glutathione Sepharose 4B (GE Healthcare) and purified protein was eluted with 50 mM Tris-HCl, 10 mM reduced glutathione, pH 8. Eluted fractions were tested for enzymatic activity by HAT assay. Active fractions were combined and used to assess inhibitor efficacy.

**Supplementary Results**

***Chemical Synthesis***

Few practical syntheses of isothiazoles have been reported [[1-6](#_ENREF_1)]. In one method, 4-arylisothiazoles were obtained by treatment of aryl-substituted β-chloroacroleins with ammonium thiocyanate (33). In a similar manner, we have prepared isothiazoles (**4** and **6**) by the reaction of 3,3-dibromoacroleins (**3a** and **3b**, respectively) with ammonium thiocyanate. Thus, treatment of 1,1-dimethoxypropanone **2a** with triphenylphosphine-carbon tetrabromide [Corey-Fuchs reaction [[7](#_ENREF_7)]] gave 3,3-dibromo-2-methylacrylaldehyde dimethyl acetal**,** which was converted into 3,3-dibromo-2-methylacrylaldehyde **3a** during chromatography on silica (Scheme 1a). Reaction of **3a** with ammonium thiocyanate gave 4-methyl-5-thiocyanatoisothiazole **4**, which arises by double displacement of bromides with thiocyanate, combination of the resulting aldehyde with ammonia and cyclization (Scheme 1a) [[8-10](#_ENREF_8)]. Treatment of aldehyde **3b**, prepared in an analogous manner to **3a**, with ammonium thiocyanate gave a mixture of compounds from which 5-thiocyanatoisothiazole **6** and 1,2-bis(isothiazol-5-yl)disulfane **7** were separated. The disulfide 1,2-bis(4-methylisothiazol-5-yl)disulfane **5** (hereinafter called NU9056) was not observed in the reaction of **3a** with ammonium thiocyanate, but was obtained on heating **4** with aqueous ammonia.

Efforts to convert **4** into bromide **1** by displacement of the thiocyanato group failed either using molecular bromine or tetrabutylammonium bromide. However, lithiation of compound **11** [[11](#_ENREF_11)] and quenching of the intermediate carbanion with bromine afforded the desired 4-methyl-5-bromoisothiazole **1**. The synthesis of isothiazole **11** was closely based on literature protocols (Scheme 1b). A sequence of dibromination of the double bond, protection of the aldehyde and elimination of hydrogen bromide was used to convert methacrolein **8** into a mixture of geometrical isomers of bromo-diethylacetal **9** [[12](#_ENREF_12)]. Hydrolysis of the acetals to aldehydes **10** was followed by treatment with ammonium thiocyanate in dimethylformamide, which gave isothiazole **11** [[13](#_ENREF_13)].

**Supplementary References**

[1]F. Wille LCAS**.** The Preparation of Isothiazole and 3-Methylisothiazole: A New Route in to the Isothiazole Series. *Angewandte Chemie International Edition in English*. 1962; 1: 335.

[2]F. Hübenett, F. H. Flock, W. Hansel*, et al.* Isothiazoles. *Angewandte Chemie International Edition in English*. 1963; 2: 714-9.

[3]Raap R**.** Concerning cis- and trans-3-thiocyanoproenal and the synthesis of isothiazole. *Canadian Journal of Chemistry*. 1966; 44: 1324-7.

[4]A. Apblett, T. Chivers**.** Synthetic applications and spectroscopic investigations of the (NSCl)_3_–SO_2_Cl_2_ system. *Canadian Journal of Chemistry*. 1990; 68: 650-4.

[5]Kang YK, Lee KS, Yoo KH*, et al.* Synthesis and biological evaluation of novel 1[beta]-methylcarbapenems with isothiazoloethenyl side chains. *Bioorganic & Medicinal Chemistry Letters*. 2003; 13: 463-6.

[6]Soledade M, Pedras C, Suchy M**.** Design, synthesis, and antifungal activity of inhibitors of brassilexin detoxification in the plant pathogenic fungus Leptosphaeria maculans. *Bioorganic and Medicinal Chemistry*. 2006; 14: 714-23.

[7]Corey EJ, Fuchs PL**.** A synthetic method for formyl®ethynyl conversion (RCHO®RCºCH or RCºCR'). *Tetrahedron Letters*. 1972; 13: 3769-72.

[8]M. Mühlstädt RBBS**.** b-Thiocyanatovinylcarbonylverbindungen. II. Darstellung von 4,5-substituierten Isothiazolen. *Journal für Praktische Chemie*. 1976; 318: 507-14.

[9]Bärbel Schulze GKSKARHH**.** Zur Oxidation von 1,2-Thiazolen: Ein einfacher Zugang zu 1,2-Thiazol-3(2<I>H</I>)-on-1,1-dioxiden. *Helvetica Chimica Acta*. 1991; 74: 1059-70.

[10]Entenmann G**.** Neue heterocyclische 8π Elektronensysteme. 1,2,3-Thiadiazine und 1,2,3-Thiadiazinium-Salze. *Synthesis*. 1973: 225.

[11]Micetich RG**.** Lithiation of 5-Membered Heteroaromatic Compounds - Methyl Substituted 1,2-Azoles, Oxadiazoles, and Thiadiazoles. *Canadian Journal of Chemistry*. 1970; 48: 2006-&.

[12]Serra S, Fuganti C**.** Baker's yeast mediated biohydrogenation of sulphur-functionalised methacrolein derivatives. Stereochemical aspects of the reaction and preparation of the two enantiomers of useful C-4 bifunctional chiral synthons. *Tetrahedron-Asymmetry*. 2001; 12: 2191-6.

[13]Pavlik JW, Tongcharoensirikul P, French KM**.** Phototransposition Chemistry of 4-Substituted Isothiazoles. The P4 Permutation Pathway. *Journal of Organic Chemistry*. 1998; 63: 5592-603.
